# Targeting Krebs-cycle-deficient renal cell carcinoma with Poly ADP-ribose polymerase inhibitors and low-dose alkylating chemotherapy

**DOI:** 10.18632/oncotarget.28273

**Published:** 2022-09-14

**Authors:** Daiki Ueno, Juan C. Vasquez, Amrita Sule, Jiayu Liang, Jinny van Doorn, Ranjini Sundaram, Sam Friedman, Randy Caliliw, Shinji Ohtake, Xun Bao, Jing Li, Huihui Ye, Karla Boyd, Rong Rong Huang, Jack Dodson, Paul Boutros, Ranjit S. Bindra, Brian Shuch

**Affiliations:** ^1^Department of Urology, David Geffen School of Medicine at UCLA, Los Angeles, CA 90095, USA; ^2^Section of Pediatric Hematology and Oncology, Department of Pediatrics, Yale University School of Medicine, New Haven, CT 06510, USA; ^3^Department of Therapeutic Radiology, Yale University School of Medicine, New Haven, CT 06510, USA; ^4^Department of Urology, West China Hospital/School of Medicine, Chengdu City, Sichuan Province, PR China; ^5^Karmanos Cancer Institute, Wayne State University, Detroit, MI 48202, USA; ^6^Department of Pathology and Laboratory Medicine, University of California, Los Angeles, CA 90095, USA; ^7^Department of Human Genetics, University of California, Los Angeles, CA 90095, USA; ^*^These authors contributed equally to this work; ^#^These authors jointly supervised this work

**Keywords:** FH, SDHB, renal cell carcinoma, PARP inhibitor, temozolomide

## Abstract

Loss-of-function mutations in genes encoding the Krebs cycle enzymes Fumarate Hydratase (*FH*) and Succinate Dehydrogenase (*SDH*) induce accumulation of fumarate and succinate, respectively and predispose patients to hereditary cancer syndromes including the development of aggressive renal cell carcinoma (RCC). Fumarate and succinate competitively inhibit αKG-dependent dioxygenases, including Lysine-specific demethylase 4A/B (KDM4A/B), leading to suppression of the homologous recombination (HR) DNA repair pathway. In this study, we have developed new syngeneic *Fh1*- and *Sdhb*-deficient murine models of RCC, which demonstrate the expected accumulation of fumarate and succinate, alterations in the transcriptomic and methylation profile, and an increase in unresolved DNA double-strand breaks (DSBs). The efficacy of poly ADP-ribose polymerase inhibitors (PARPis) and temozolomide (TMZ), alone and in combination, was evaluated both *in vitro* and *in vivo*. Combination treatment with PARPi and TMZ results in marked *in vitro* cytotoxicity in *Fh1*- and *Sdhb*-deficient cells. *In vivo*, treatment with standard dosing of the PARP inhibitor BGB-290 and low-dose TMZ significantly inhibits tumor growth without a significant increase in toxicity. These findings provide the basis for a novel therapeutic strategy exploiting HR deficiency in FH and SDH-deficient RCC with combined PARP inhibition and low-dose alkylating chemotherapy.

## INTRODUCTION

Alterations in Krebs cycle genes that encode Fumarate Hydratase (*FH*) and Succinate Dehydrogenase (SDH) are associated with cancer predisposition syndromes [1, 2]. Germline *FH* mutations result in Hereditary Leiomyomatosis and Renal Cell Cancer (HLRCC) syndrome, characterized by cutaneous and uterine leiomyomas and renal cell carcinomas with unique nucleolar features [3]. Renal tumors associated with FH mutations tend to occur earlier in life (median age ~35) and can have an aggressive disease course [4, 5]. Germline mutations in the *SDH* genes (*A, B, C, D, or AF2*) cause a Hereditary Paraganglioma and Pheochromocytoma (HPGL/PCC) syndrome, with a predisposition towards paragangliomas, pheochromocytomas, gastrointestinal stromal tumors, and renal cell carcinomas (RCC) [6]. The majority of SDH-deficient renal cancers involve mutations in *SDHB,* and these tumors can similarly behave aggressively with early age of onset [7, 8]. Unfortunately, for both advanced stage FH- or SDH-deficient RCC, there are limited treatment options, creating a significant unmet need [9].

FH and SDH are key elements of the Krebs cycle, and loss of function can cause accumulation of the metabolites fumarate and succinate, respectively [10]. Besides the characteristic metabolic changes, such as impairment in oxidative phosphorylation, succinate and fumarate, when aberrantly accumulated, both act as oncometabolites that competitively inhibit αKG–dependent dioxygenases, dysregulating DNA methylation and histone modification [11, 12]. We previously demonstrated that FH and SDHB deficient tumors have elevated levels of fumarate and succinate sufficient to suppress the homologous recombination (HR) DNA-repair pathway through inhibition of Lysine-specific demethylase 4B (KDM4B), which subsequently results in aberrant hypermethylation of histone 3 lysine 9 (H3K9) at loci surrounding DNA breaks. Without this trimethylation signal to execute proper HR, tumor cells are vulnerable to synthetic-lethal targeting with PARP inhibitors [13]. To further potentiate the effect of PARP inhibitors, combinations with other agents have been explored in other tumor types. Temozolomide (TMZ), an alkylating agent, mediates its cytotoxic effect by attaching methyl groups to DNA, with the N3-MetA and N7-MetG adducts requiring repair via the base excision repair (BER) pathway in a process involving PARP [14]. Thus, the combination of PARP inhibitors (PARPis) with TMZ has been shown to increase TMZ-induced cytotoxicity [15].

In this study, we sought to examine the activity of combined TMZ and PARPi in Krebs-cycle-deficient renal cancer models. *Fh1* and *Sdhb* are the murine counter part of human *FH* and *SDHB*, respectively. Using newly developed Fh1 and Sdhb deficient syngeneic mouse models, we demonstrate that oncometabolite-induced HR defects can be leveraged with PARPi treatment to enhance sensitivity to low-dose TMZ in Krebs-cycle-deficient renal cancer.

## RESULTS

### Fh1- and Sdhb-deficient cells accumulate fumarate and succinate respectively and display decreased oxygen consumption rate

**Figure 1:**
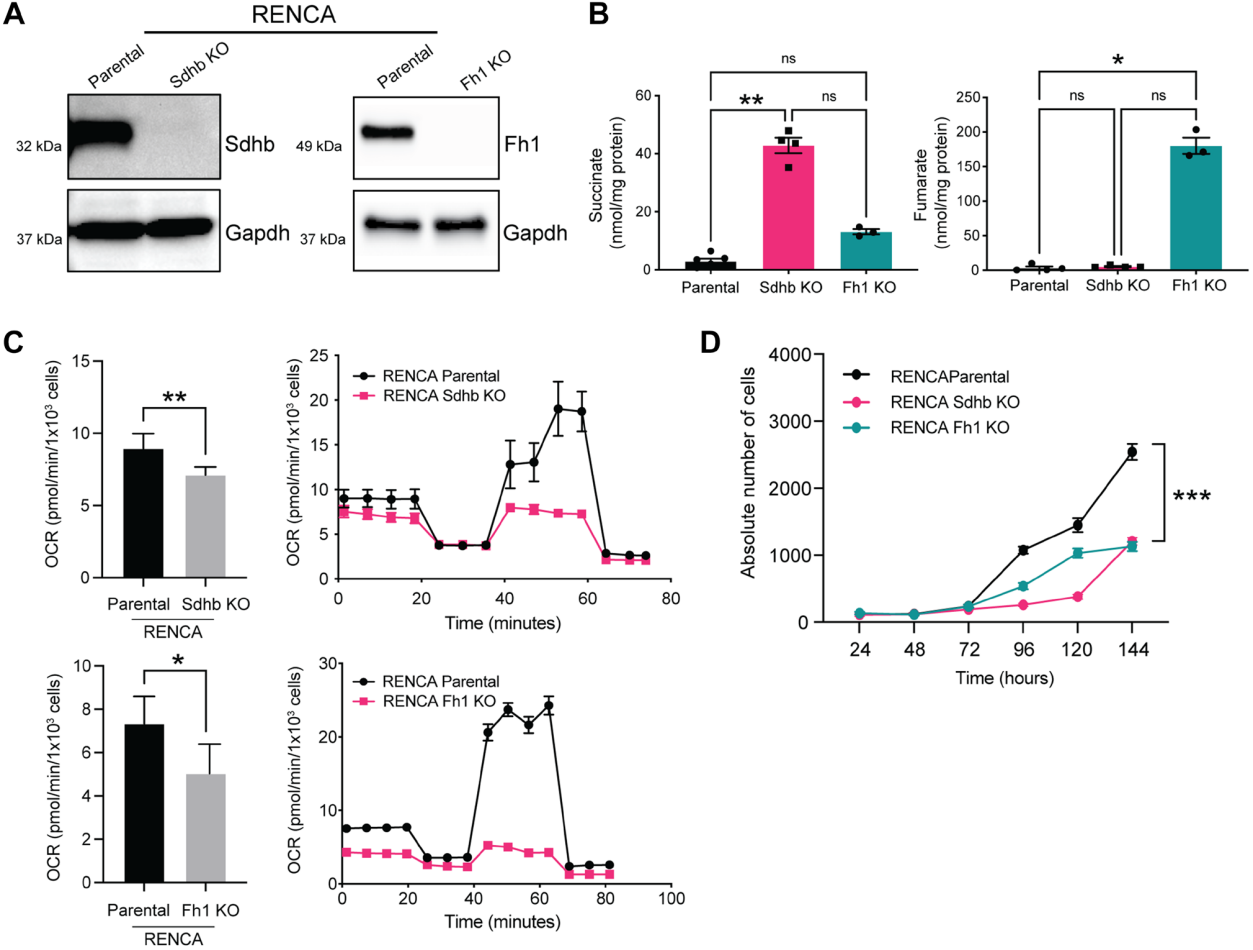
Fh1- and Sdhb-deficient cells accumulate fumarate and succinate respectively and display decreased oxygen consumption rate. (**A**) Western blot confirming CRISPR/Cas9 mediated KO of Sdhb (left panel) and Fh (right panel). GAPDH was used as a loading control. (**B**) Functional validation of succinate (left panel) and fumarate (right panel) levels by LC-MS in isogenic cell lines (*n* = 3). (**C**) Seahorse assay showing altered OCR in Fh1-KO (top panel) and Sdhb-KO (bottom panel). (**D**) Proliferation rates of mutant vs. parental cells (*n* = 3). Error bars represent means ± SEM. ^***^ *P* < 0.001, ^**^ *P* < 0.01, ^*^ *P* < 0.05.

We engineered isogenic Fh1 and Sdhb knockout RENCA cells using CRISPR/Cas9 with Fh1 and Sdhb knockout confirmed by western blot (Figure 1A). Fumarate and succinate accumulation has been demonstrated in FH-deficient and SDH-deficient tumors and cell lines, respectively [10, 16]. Fumarate and succinate levels were measured in cell pellets by liquid chromatography-mass spectrometry (LC-MS). In line with data from patient tumor samples, we found significantly elevated levels of fumarate and succinate in Fh1 and Sdhb-KO cells (Figure 1B).

We next performed Seahorse metabolic flux assay to determine whether Fh1 and Sdhb mutations cause Krebs cycle dysfunction and alter metabolism. Compared to RENCA parental cells, oxidative phosphorylation (OXPHOS), as measured by the oxygen consumption rate (OCR), was decreased in Fh1-KO and Sdhb-KO cells (Figure 1C). Conversely, both models demonstrated a shift toward aerobic glycolysis, as measured by the extracellular acidification rate (ECAR) (Supplementary Figure 1A). We also transfected Fh1-KO cells with an open reading frame (ORF) of Fh1 and confirmed normalization of fumarate levels (Supplementary Figure 1B and 1C). Fh1-rescued cells showed recovered OCR , suggesting that the observed metabolic perturbations were due to loss of Fh1 and not secondary to off-target effects (Supplementary Figure 1D).

Fh1- and Sdhb-KO cells also showed altered morphology *in vitro*. Compared to RENCA parental cells, which show a well-described epithelial-like morphology [17], Fh1- and Sdhb-KO cells are elongated, with fibroblast-like morphology and reduced cell-cell contact (Supplementary Figure 1E). Fh1- and Sdhb-KO cells also showed a decreased proliferation rate *in vitro* when compared to RENCA parental cells (Figure 1D). Fh1- and Sdhb-KO cells also showed increased expression of key genes related to glycolysis and decreased expression of key genes related to Krebs cycle by RNA-seq, as expected (Supplementary Figure 1F, 1G and 1H).

### Sdhb and Fh1-KO cells differentially express genes related to cell-cycle and DNA repair

**Figure 2:**
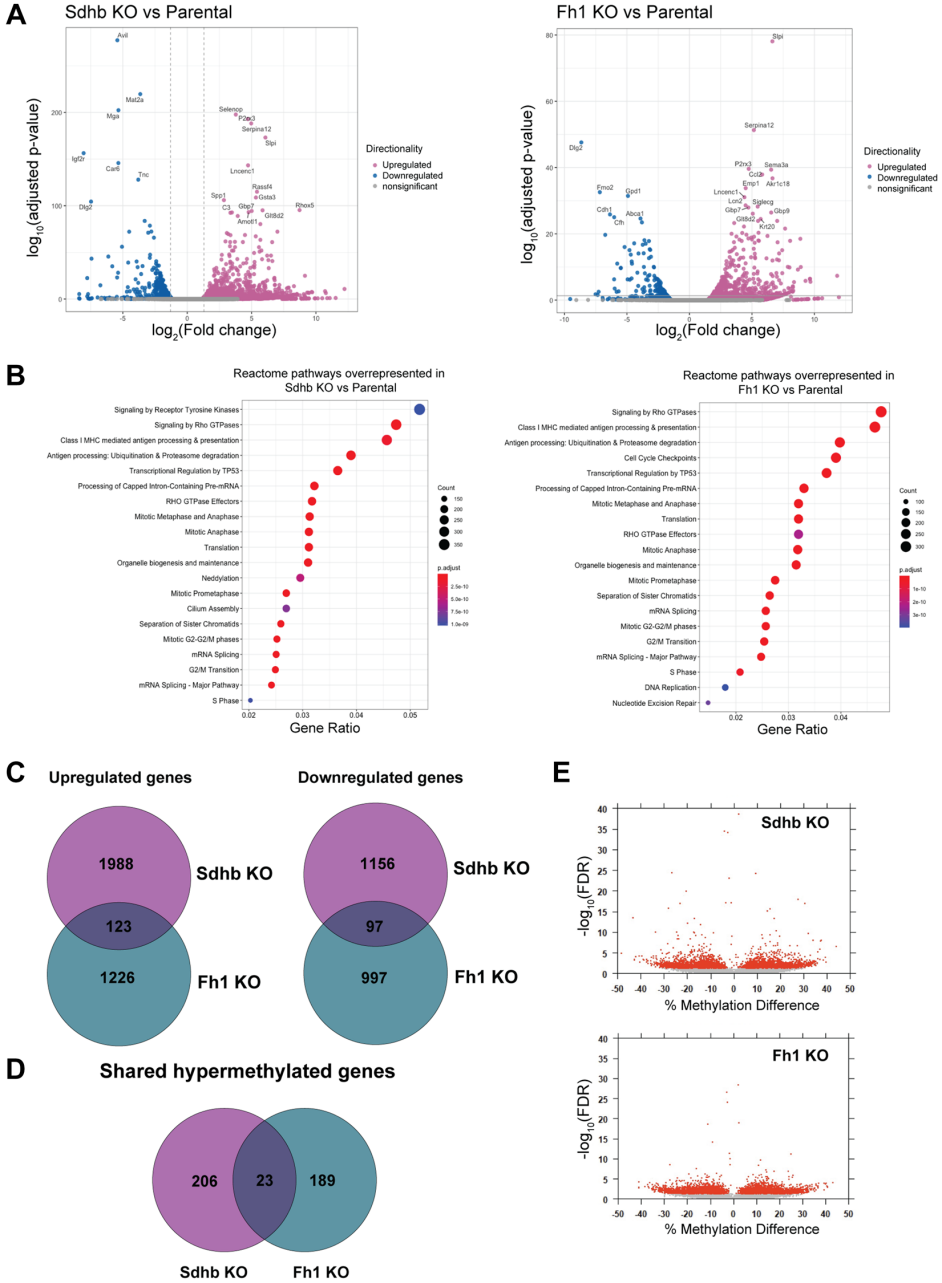
Gene expression profile of engineered cell lines by RNA-seq. (**A**) The volcano plot displays the significant up-regulated (pink) and down-regulated (blue) genes in the Sdhb-KO (left panel) and Fh1-KO (right panel) cells compared to the parental cells. Top 20 DGE’s are labelled. (**B**) Reactome over-representation analysis showing top 20 over-represented biological Reactome pathways for DGE’s associated with Sdhb-KO (left panel) and Fh1-KO (right panel). (**C**) Venn diagram showing upregulated genes and downregulated genes shared between Sdhb-KO and Fh1-KO. (**D**) Venn diagram showing the hypermethylated CpG island shared between Sdhb-KO and Fh1-KO. (**E**) Logistic regression volcano plots. Each point represents a differentially methylated CpG site, with red points exhibiting FDR adjusted *p*-Value < 0.05.

To further characterize our model, we performed RNA-seq in Sdhb-KO, Fh1-KO and parental cells to identify differentially expressed genes (DEGs). The top 20 DGE’s are annotated in the volcano plot for Sdhb-KO (Figure 2A, left panel) and Fh1-KO (Figure 2A, right panel). We then used clusterProfiler [18] to perform over-representation gene ontology (GO) analysis and assess known signaling reactome pathways enriched on the list of DEGs identified with foldchange >1 and *p* < 0.05 cutoffs. Reactome pathway analysis indicated that several pathways associated with cell-cycle were enriched in both Sdhb-KO and Fh1-KO DEGs (Figure 2B). Also, GO over-representation analysis showed that DNA repair was the top biological process (BP) in both Sdhb-KO and Fh1-KO DEGs (Supplementary Figure 2). In total, we found a total of 3364 genes differentially expressed in Sdhb-KO cells compared to parental cells of which 2111were upregulated and 1253 were downregulated. We found a total of 2443 genes differential expressed in Fh1-KO cells compared to parental cells of which 1349 were upregulated and 1094 were downregulated, with fold change >1 and <–1, and padj <0.05 cutoffs. There were 123 upregulated genes and 97 downregulated genes that were shared between Sdhb-KO and Fh1-KO (Figure 2C and Supplementary Tables 1 and 2).

### Fh1-KO and Sdhb-KO cells display differential methylation profiles

It has been shown that mutations in FH and SDHB are associated with hypermethylator phenotypes [19, 20]. We performed whole genome bisulfite sequencing to identify the impact on DNA methylation by loss of Fh1 and Sdhb. Global methylation profiles were similar between Fh1-KO and Sdhb-KO cells when compared to parental cells with an average methylation of 40–60% (Supplementary Figure 3A). However, a principal component analysis (PCA) showed a clear separation between the KO and parental cells, suggesting significant locus-specific differential methylation (Supplementary Figure 3B). Next, we analyzed differential methylation hits located within CpG islands, CpG shores (defined as extending 2kb out wards from CpG island), and CpG shelves (extending 2kb outward from CpG shores) in both Fh1-KO and Sdhb-KO relative to the parental cells. In total, 212 loci in Fh1-KO, and 229 loci in Sdhb-KO were identified to be hyper-methylated. Only 23 loci were shared with Fh1-KO and Sdhb-KO cells (Figure 2D and 2E, Supplementary Tables 3 and 4).

### Krebs-cycle-deficient cells are tumorigenic *in vivo* and have increased protein succination

**Figure 3:**
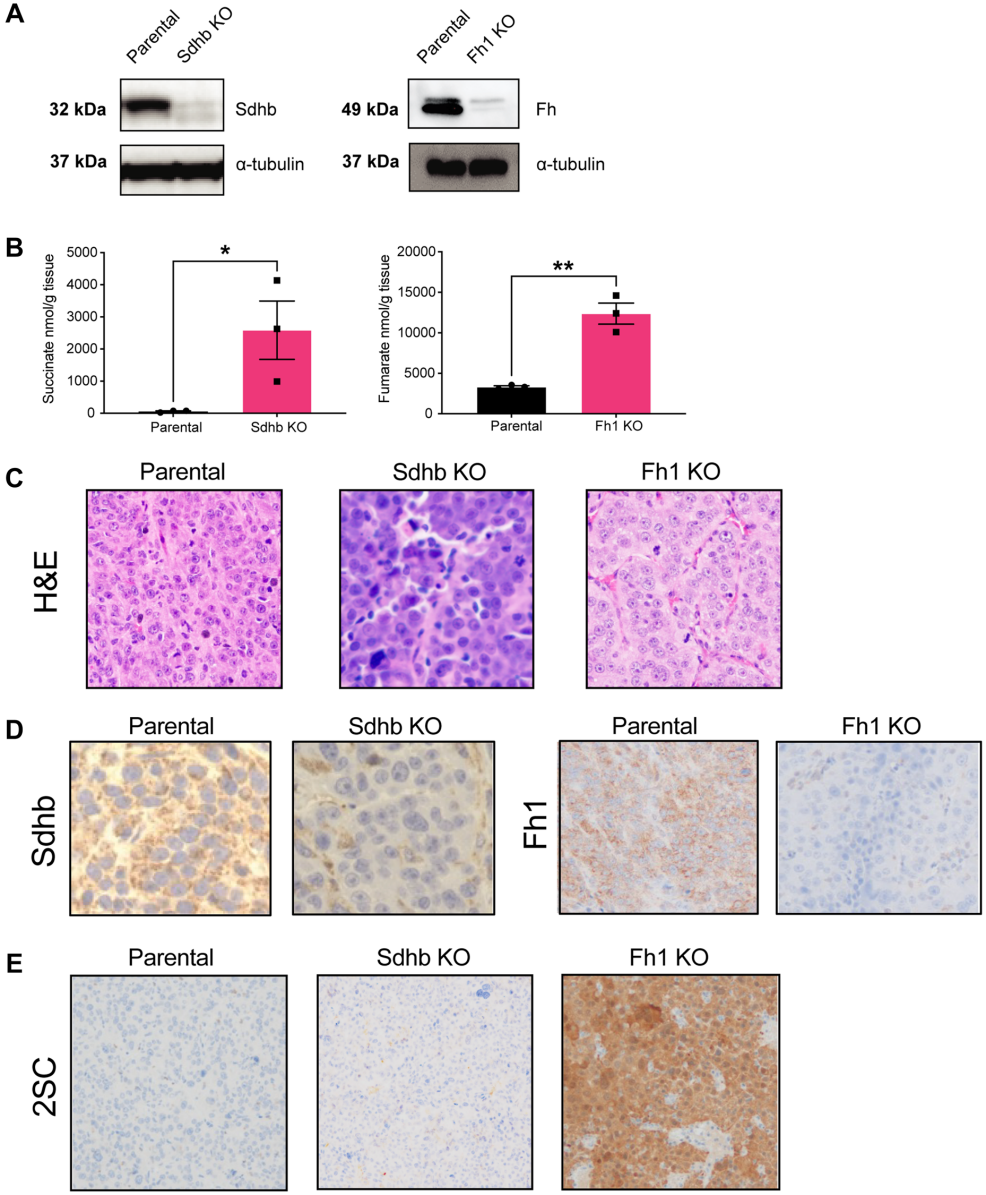
Krebs-cycle-deficient cells are tumorigenic *in vivo* and have increased protein succination. (**A**) Western blot from tumor lysate showing that syngeneic tumors retain loss of Sdhb (left panel) and Fh1 (right panel) expression, respectively. a-tubulin was used as loading control. (**B**) LC-MS analysis showing that Sdhb- and Fh1-deficient tumors retain succinate (left panel) and fumarate (right panel) accumulation *in vivo* (*n* = 3). (**C**) Representative H&E staining showing altered tumor morphology in Fh1 and Sdhb-deficient tumors. (**D**) Representative immunohistochemistry showing loss of Sdhb (left panel) and Fh1 (right panel) expression in tumor tissue. (**E**) Representative immunohistochemistry showing increased expression of 2SC in Fh1 KO tumor. Error bars represent means ± SEM. ^***^ *P* < 0.001, ^**^ *P* < 0.01, ^*^ *P* < 0.05.

We sought to determine the *in vivo* characteristics of Fh1- and Sdhb-KO cells. Similar to RENCA parental cells, both Fh1- and Sdhb-KO cells reliably form flank tumors. Harvested tumors maintain Fh1- and Sdhb-KO and demonstrate accumulation of fumarate and succinate by LC-MS (Figure 3A and 3B). Histologically, RCC associated with FH and/or SDHB mutations can demonstrate a spectrum of architectural patterns, often with characteristically large nuclei and prominent inclusion-like eosinophilic nucleoli with a surrounding perinucleolar halo [21, 22]. In this study, compared to tumors formed by RENCA parental cells, harvested Fh1-KO tumors displayed altered morphology characterized by enlarged cells with large nuclei, prominent eosinophilic nucleoli, and perinucleolar halos (Figure 3C), similar to the characteristic features of HLRCC tumors [21]. Sdhb-KO tumor cells also demonstrated altered morphology with more nuclear overlapping, a higher nuclear:cytoplasmic ratio, amphophilic to basophilic cytoplasm, anaplasia, and increased cellular pleomorphism (Figure 3C).

Immunostaining for both Fh1, Sdhb and S-(2-succino)-cysteine (2SC) was performed on formalin-fixed paraffin-embedded (FFPE) tumor sections confirming loss of Fh1 and Sdhb (Figure 3D). 2SC staining was used as an additional means of validating Fh1 inactivation, as 2SC production is caused by elevated intracellular fumarate reacting with the cysteine residues of many proteins leading, a process known as succination [23]. 2SC was strongly positive in Fh1-KO tumors, consistent with HLRCC patient tumors (Figure 3E). Meanwhile, 2SC was not increased in both Sdhb-KO tumors and RENCA parental tumors (Figure 3E).

### Fh1 and Sdhb-deficient cells have increased DNA damage and marked PARP-inhibitor and temozolomide sensitivity *in vitro*

**Figure 4:**
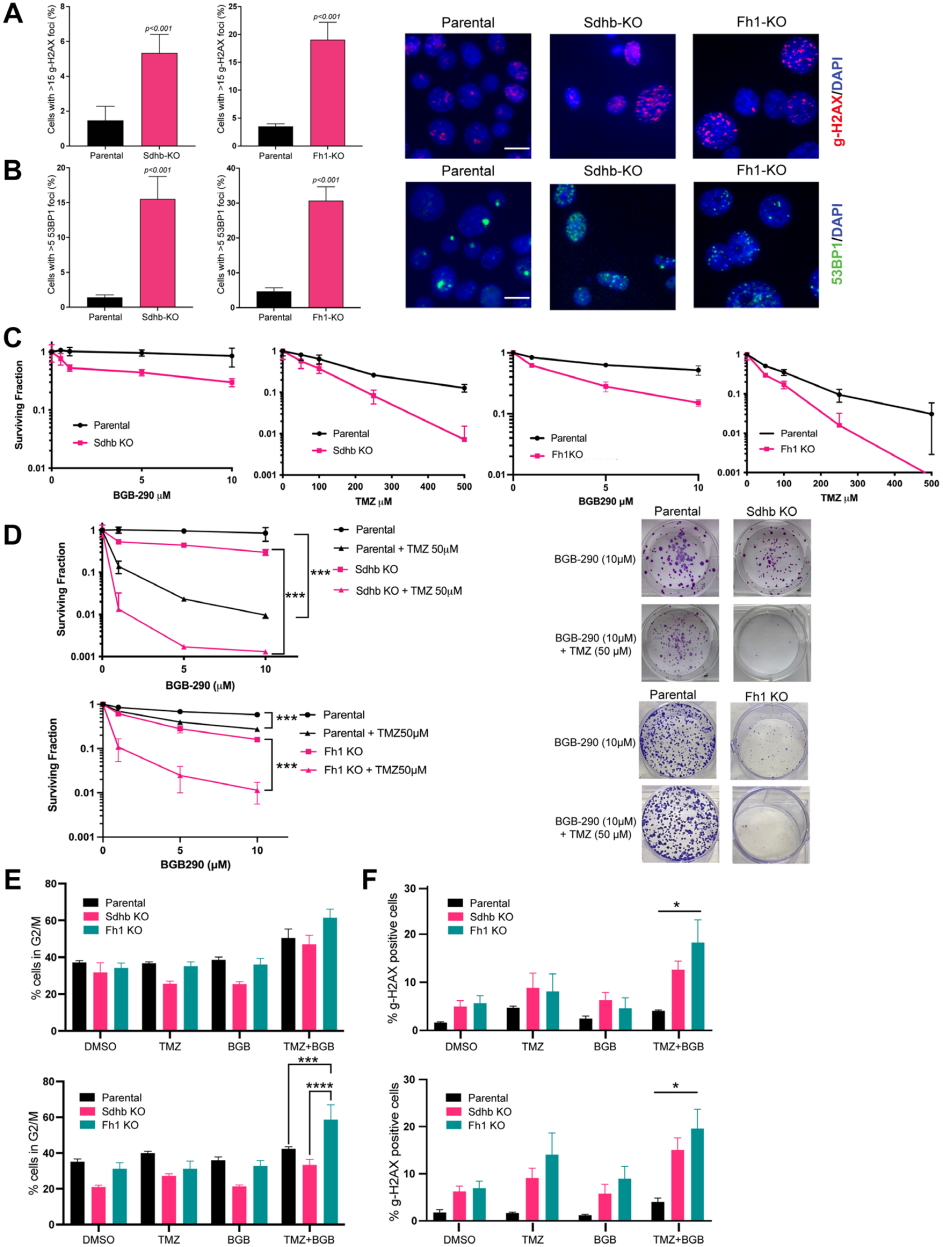
Fh1 and Sdhb-deficient cells have increased DNA damage and marked PARP-inhibitor and temozolomide sensitivity *in vitro*. (**A**) γ-H2AX foci quantification (left panel) and representative immunostaining (right panel) in RENCA parental, Sdhb-KO and Fh1-KO cells. Cells were fixed and stained with γ-H2AX (red) and counterstained with DAPI (blue). Cells with more than 15 γ-H2AX foci were counted in 10–12 distinct fields. The images shown were acquired using a 40× objective lens. The scale bar is 10 μm (*n* = 2). (**B**) 53BP1 foci quantification (left panel) and representative immunostaining (right panel) in RENCA parental, Sdhb-KO and Fh1-KO cells. Cells were fixed and stained with 52BP1 (green) and counterstained with DAPI (blue). Cells with more than 5 53BP1 foci were counted in 10–12 distinct fields. The images shown were acquired using a 40× objective lens. The scale bar is 10 μm (*n* = 2). (**C**) Quantification of clonogenic survival assays of RENCA parental vs. Sdhb-KO and Fh1-KO cells treated with monotherapy BGB-290 (left panel) and monotherapy temozolomide (right panel) for 12–14 days (*n* = 3). (**D**) Quantification (left panel) and representative images (right panel) of clonogenic survival assays of RENCA parental vs. Sdhb-KO and Fh1-KO cells treated with a dose range of BGB-290 in combination with 50 μM temozolomide for 12-14 days (*n* = 3). (**E**) Cell-cycle analysis of RENCA parental (WT), Sdhb-KO (JL1) and Fh1-KO (B4) cells treated with DMSO control, BGB-290 (1μM), TMZ (50 μM) or combined BGB-290 and TMZ for 24 hours (top panel) or 48 hours (bottom panel) (*n* = 2). (**F**) Quantification of γ-H2AX expressing cells. RENCA parental (WT), Sdhb-KO (JL1) and Fh1-KO (B4) cells were treated with DMSO control, BGB-290 (1 μM), TMZ (50 μM) or combined BGB-290 and TMZ for 24 (left panel) and 48 hours (right panel) (*n* = 3). Error bars represent means ± SEM. ^***^ *P* < 0.001, ^**^ *P* < 0.01, ^*^ *P* < 0.05.

Next, we sought to assess the intrinsic double-strand break (DSB) repair capability of our models by evaluating markers of DNA damage at baseline. Phosphorylated Histone 2A variant H2AX (γ-H2AX) and p53-binding protein 1 (53BP1) accumulate at chromatin surrounding DSB’s as part of the cellular response to DNA DSBs and are elevated in HR-deficient cells [13, 24]. We performed immunofluorescence (IF) foci studies in log-phase cells and found that Fh1 or Sdhb1 knockout resulted in elevation of γ-H2AX and 53BP1 foci compared to parental (Figure 4A and 4B).

We hypothesized that the HR defect conferred by Fh1 or Sdhb deficiency might increase sensitivity to other DNA-damaging agents, such as TMZ, an alkylating chemotherapy. We performed clonogenic survival assays and found that Fh1 and Sdhb deficient cells displayed increased sensitivity to single agent treatment with PARPi (BGB-290) and TMZ monotherapy relative to RENCA parental cells (Figure 4C). Fh1-rescued cells showed the same minimal sensitivity to PARPi as parental RENCA cells, and parental RENCA cells were used as negative controls in subsequent experiments (Supplementary Figure 4A).

Next, we tested for the ability of TMZ to potentiate the *in vitro* activity of BGB-290. Cells were treated with doses of BGB-290 ranging from 1 μM to 10 μM, in the presence or absence of 50 uM TMZ. Combined treatment significantly enhanced cytotoxicity with the most pronounced effect in Fh1 and Sdhb deficient cells (Figure 4D).

Next, we sought to examine the mechanistic basis for increased cytotoxicity with combination treatment *in vitro*. Treatment with BGB-290 and TMZ induced G2/M arrest after 24 hours in Fh1-KO and Sdhb-KO cells as well as RENCA parental cells (Figure 4E, top panel). After 48 hours of treatment, there remained a persistent and significant G2/M arrest in Fh1 KO cells (Figure 4E, bottom panel). We then assessed for DNA damage after 24 and 48 hours of drug treatment by measuring γ-H2AX via flow cytometry. Consistent with IF data, Fh1- and Sdhb-KO cells harbored an increase in γ-H2AX with vehicle control, which was further increased with treatment (Figure 4F).

### Sdhb deficiency confers sensitivity to combined PARP-inhibitor and low-dose TMZ *in vivo*

**Figure 5:**
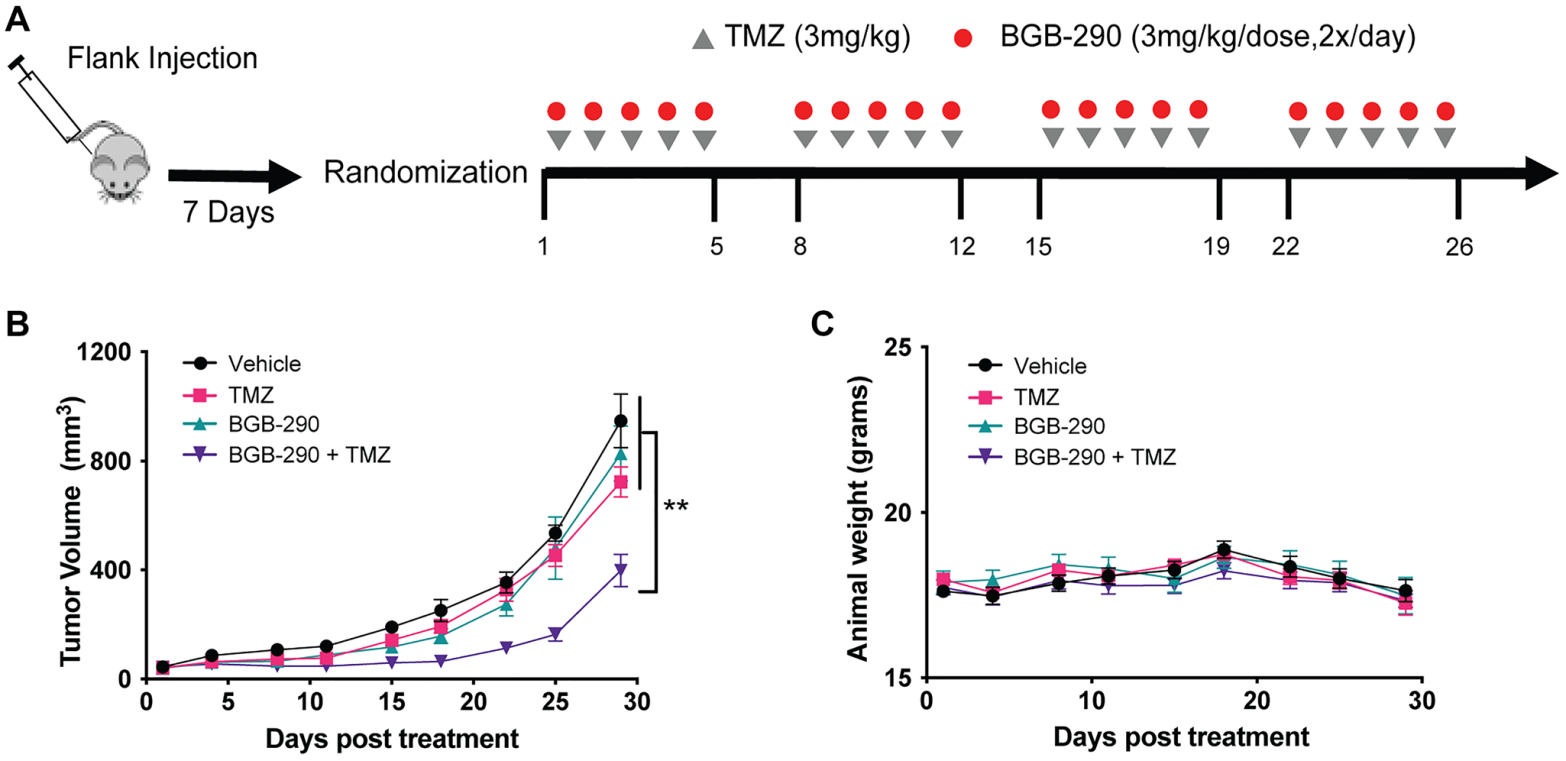
Sdhb deficiency confers sensitivity to combined PARP-inhibitor and low-dose TMZ *in vivo*. (**A**) Experimental schema of *in vivo* treatments. RENCA Sdhb-KO cells were injected subcutaneously into the flank of BALB/c mice. Seven days after injection, tumors were measured and mice were randomized into four-arm treatment groups. Treatment was initiated when tumors reached an average size of 40–100 mm^3^. (**B**) Mice carrying flank tumors of Sdhb-KO cells were treated with no treatment (*n* = 9), TMZ alone (3 mg/kg) (*n* = 10), BGB-290 (3 mg/kg/dose, 2x/day) (*n* = 10), or TMZ (3 mg/kg) and BGB-290 (3 mg/kg/dose, 2x/day) (*n* = 9). Mice were treated in 5-day cycles for a total of 4 cycles. Data are represented as mean ± SEM. (**C**) Mean body weight of mice during Sdhb-KO flank tumor experiment. Data are represented as mean ± SEM. *P* values were calculated using two-way ANOVA. ^***^ *P* < 0.001, ^**^ *P* < 0.01, ^*^ *P* < 0.05.

Lastly, we performed a limited *in vivo* study in our Sdhb deficient RENCA flank model as a proof-of-concept to validate *in vitro* observations. Due to toxicities that have been encountered in clinical trials [25, 26], we evaluated standard doses of PARPi combined with a low dose of TMZ. Typically, a TMZ dosing range of 25–50mg/kg is employed in pre-clinical studies and corresponds to a human equivalent dose (HED) of 75–150 mg/m^2^, respectively [27]. In a pilot study, we found that low-dose TMZ alone at 3 mg/kg/dose showed minimal *in vivo* efficacy (Supplementary Figure 4B and 4C) and therefore this dosage was chosen for subsequent *in vivo* studies (Figure 5A).

Consistent with *in vitro* data, the combination of standard dose BGB-290 and low-dose TMZ resulted in significantly delayed tumor progression compared to single-agent treatment in a Sdhb-deficient RENCA model (Figure 5B). This delay in tumor progression was not accompanied by any significant increase in toxicity, as evaluated by change in animal body weight (Figure 5C).

## DISCUSSION

Mutations of the Krebs-cycle genes that encode fumarate hydratase (*FH*) and succinate dehydrogenase (*SDH*) are associated with cancer predisposition syndromes, including HLRCC and SDH PGL/PCC, both characterized by the propensity to develop aggressive tumors, including RCC [1, 2]. In this study, we established new murine models of Krebs-cycle-deficient RCC that mimic human tumors and we demonstrate that combined PARPi and low-dose TMZ demonstrates significant anti-tumor activity.

The hallmarks of Krebs-cycle enzyme deficient syndromes were recapitulated in our model, including loss of enzyme function in parallel with fumarate and succinate metabolite accumulation in each respective KO line. As expected with perturbations of the Krebs-cycle from loss-of-function of either Fh1 or Sdhb, we demonstrated a decrease in oxidative phosphorylation and a shift to glycolytic metabolism [28]. For Fh1 KO cells, the high level of fumarate accumulation resulted in strong 2SC staining consistent with the aberrant succination of cellular proteins as is also documented in HLRCC tumor samples from patients [21]. Oncometabolite accumulation also disrupts the activity of a superfamily of α-KG-dependent dioxygenases, such as the Ten-eleven translocation (TET) family that are critical to DNA demethylation, inducing profound epigenetic reprogramming [29, 30]. Our methylation analysis demonstrates a unique methylation profile that is distinct between wild-type and matched isogenic lines, in line with epigenetic instability from hypermethylation [19, 20]. While global methylation did not change it is possible that these epigenetic changes are time dependent. We previously demonstrated that elevated levels of fumarate and succinate suppress HR through inhibition of the KDM4B and subsequent aberrant hypermethylation of histone 3 lysine 9 (H3K9) at loci surrounding DNA breaks. This hypermethylation serves to mask a local H3K9 trimethylation signal that is essential for the proper execution of HR and leads to synthetic lethality with PARP inhibitors [13]. In line with previous findings, we showed that Fh1 or Sdhb knockout resulted in increased levels of unrepaired DSBs, as measured by γ-H2AX and p53BP1 foci. Furthermore, we found alterations in the transcriptomic profile of Fh1 and Sdhb knockout cells with reactome pathway analysis indicating the expected enrichment of genes involved in cell-cycle and DNA repair pathways.

Based on our previous work, we hypothesized that an oncometabolite-induced DNA repair defect can be further exploited with the addition of DNA damaging agents, such as alkylating chemotherapy. We focused on TMZ due to the role of PARP in BER and the potential synergy with PARPi. PARP-trapping on DNA single-strand breaks is fundamental to the synergistic effect of PARPi and TMZ combination therapy [31, 32]. BGB-290, a potent PARP trapper, demonstrated significant *in vitro* cytotoxicity in Fh1- and Sdhb-KO cells when combined with TMZ, resulting in G2/M cell-cycle arrest along with increased γ-H2AX in Krebs-cycle-deficient cells. Taken together, these findings suggests that elevated levels of DNA damage partially mediate increased sensitivity to treatment.

Clinical trials have investigated PARPi and TMZ combination therapy in a variety of cancers but have been hampered by toxicity at standard TMZ dosing [25, 26]. For pre-clinical murine studies, TMZ is typically used at 25–50 mg/kg, which corresponds to the standard 75–150 mg/m^2^ used clinically. In this study, we demonstrated decreased tumor growth with a significantly lower TMZ dose of 3 mg/kg, which corresponds with a HED of only 9 mg/m^2^, when combined with the BGB-290. These findings suggest that combined PARP inhibition and low-dose alkylator therapy may be a novel therapeutic strategy that may have an improved safety profile that warrants further testing in Krebs-cycle-deficient RCC.

Lastly, immune checkpoint blockade has become an important modality in the treatment of advanced renal cell carcinoma [33]. However, the pre-clinical testing of these agents is limited by the availability of suitable syngeneic animal models with an intact immune system. In the setting of other tumor associated Krebs-cycle mutations, such as IDH1/2, the build-up of oncometabolites has been shown to alter the immune microenvironment and potentially modulate response to immunotherapy [34]. Additionally, PARP inhibition in the setting of HR-deficiency has been shown to potentiate anti-tumor immunity and improve response to Programmed Cell Death Protein-1 (PD-1) blockade [35]. Here, we developed new RCC models using the syngeneic RENCA cell line that will facilitate the study of immunotherapies and the anti-tumor immune response in the context of Krebs-cycle mutations and oncometabolite accumulation.

## MATERIALS AND METHODS

### Cell culture and proliferation assay

RENCA was obtained from the American Type Culture Collection (ATCC). The RENCA, RENCA Fh1-KO, RENCA Sdhb-KO cells were cultured in a humidified atmosphere at 37°C in RPMI1640 medium with 10% fetal bovine serum and non-essential amino acids (0.1 mM extra), sodium pyruvate (1 mM extra), L-glutamine (2 mM extra). To measure *in vitro* proliferation, cells were plated in 96 well dishes at 500 cells/well in triplicate. Each day, on 6 consecutive days, cells were fixed with 3.7% paraformaldehyde and stained with Hoechst (1 μg/ml). Plates were then imaged on a Cytation 3 automated imager (BioTek), and cells were counted using CellProfiler (http://cellprofiler.org/).

### CRISPR/Cas9 genomic editing and plasmids

#### Sdhb

CRISPR/Cas9 genomic editing was performed in RENCA cells using expression of both Cas9 (Addgene #43861) and a gRNA plasmid construct pmU6-gRNA (Addgene #53187) gifted by Dr. Keith Joung [36] and Dr. Charles Gersbach respectively [37]. *SDHB* gRNA sequences can be found in Supplementary Table 5 and were synthesized, annealed, and ligated into the gRNA plasmid. Both constructs were then co-transfected into RENCA cells via nucleofection (Lonza), and the cells were incubated for 72 hours prior to harvest and isolation. Isolated clones were generated through a limited dilution approach and were grown up from single cells in individual wells of a 96-well plate. Clones were screened for Sdhb- knock out by western blotting as described below. To analyze different alleles of the knock-out clones, target regions were amplified by PCR (Supplementary Table 5) and amplicons were cloned into the TOPO PCR cloning kit (Thermo Fisher Scientific) and transformed into STBL3 chemically competent cells. Bacterial colonies with plasmids harboring inserts were selected by colony direct PCR Sanger sequence after Exonuclease I and FastAP alkaline phosphatase treatment [37]. Heterozygous clones were re-transfected with Cas9 and gRNA plasmid construct pmU6-gRNA to obtain the SDHB-KO cloned verified by western blotting and sequencing.

#### Fh1

Genomic editing was performed in RENCA cells using CRISPR/Cas9 KO Plasmid (m) (sc-420348, Santa Cruz Biotechnology) and fumarate hydratase HDR Plasmid (m) (sc-420348-HDR, Santa Cruz Biotechnology). RENCA cells were plated on 6-well plates and cultured overnight and the next day transfected with both plasmids using Fugene 6 (Promega) as described by the manufacturer, gRNA target sequence can be found in Supplementary Table 6. Forty-eight hours post transfection, cells were selected with 1 μg/ml of puromycin (Sigma-Aldrich) for 1 week. RFP (Red Fluorescent Protein) positive cells were sorted using BD FACSAriaIII cell sorter (Beckton, Dickinson and Company) as Fh1 knockout polyclonal cells. Isolated clones were generated through a limited dilution approach on 96-well plates, and cloned cells were collected for protein expression analysis.

### Generating Fh1-rescued cell line

The Fh1 open reading frame was cloned into pLneti6/V5-p53_wt p53 (Addgene plasmid #22945). The resulting plasmid was pLenti6/V5-Fh1. HEK293T cells were transfected with the pLenti6/V5-Fh1, pRSV-Rev (Addgene plasmid #12253), pMDLg/pRRE (Addgene plasmid #12251) and pCMV-VSV-G (Addgene plasmid #8454). Forty-eight hours post-transfection, the supernatant was collected, then filtered at 0.2 μm.

RENCA Fh1-KO cells were transduced with lentivirus with 8 μg/ml polybrene. Sixteen hours after transduction, medium was replaced with fresh medium and 24 hours later, medium was replaced with fresh medium containing 4 μg/ml blasticidin.

### Western blotting

Whole cell lysates were prepared using RIPA buffer (Cell Signaling Technology) with 1x protease and phosphatase inhibitor (78442, ThermoFisher Scientific). Lysate protein concentration was quantified via Bradford assay (Bio-Rad Bradford reagent #5000006), and a standard quantity of protein was loaded into each lane. A total of 10–30 μg of protein was resolved using sodium dodecyl sulfate-polyacrylamide (SDS-PAGE) electrophoresis. Proteins were transferred to a nitrocellulose or polyvinylidene fluoride (PVDF) membrane and blocked with 5% nonfat milk or 5% bovine serum albumin (BSA) in TBST (10 mM Tris-HCl, 100 mM NaCl, 0.1% Tween 20). Membranes were then incubated with primary antibody at 4°C overnight at a 1:1,000 dilution and incubated with horseradish-peroxidase linked secondary antibody at 1:5,000–10,000 dilution. Antibodies used include the FH antibody (#4567S Cell Signaling Technology), GAPDH antibody (#3683 Cell Signaling Technology or ProteinTech #HRP-60004), SDHB antibody (Abcam #ab14714, Sigma #HPA002868), and a-tubulin (Abcam #ab4074, CST #2144S). Immunoblot exposure was performed using Clarity Western ECL substrate (BioRad) and blots were imaged with the ChemiDoc Touch MP (Bio-Rad Laboratories Inc).

### Immunofluorescence

A total of 25,000 cells were seeded on glass chamber slides and treated with drugs for 24 hours. Cells were fixed with 3.7% paraformaldehyde. Cells were then washed with PBS and permeabilized with 0.2% Triton-PBS. Cells were incubated with primary antibody (anti-γ-H2AX, Millipore Sigma, #05-636) or (anti-53BP1, Cell Signaling #2675) overnight at 4°C and secondary antibody (Alexa Flour 674 and Alexa Flour 594, respectively) at room temperature for 1 hour. Cells were stained with DAPI and analyzed on a Keyence BZ-X800.

### LC-MS analysis

Concentrations of succinate and fumarate in frozen cell and tumor tissue samples were determined by LC-MS/MS, as described previously with modification [38]. In brief, frozen cell pellets were lysed under sonification and frozen tissue was homogenized in water (at 2000 g). Cell or tissue homogenate was spiked with 6 μL internal standard solution containing succinate-d_6_, fumarate-1,4-^13^C_2_ 2,3-d_2_, and extracted by protein precipitation twice with ice-cold methanol and 80% methanol. The supernatant was combined and dried in a CentriVap Concentrator (Labconco) at 10°C. The residue was reconstituted in water, and 5 μL of the supernatant was injected into the AB Sciex QTRAP6500 LC-MS/MS system (Sciex). The chromatographic separation was performed on a Phenomenex Synergi™ Polar-RP column (150 × 2 mm, 4 μm) (Phenomenox). Column eluents were monitored under a negative electrospray ionization mode using the multiple reaction monitoring (MRM). Succinate, fumarate, and their respective stable isotope-labeled internal standards were monitored at their respective mass transitions.

### Measurements of oxygen consumption and extracellular acidification

The XF96 extracellular flux analyzer (Seahorse Bioscience) was used to measure oxygen consumption rate (OCR) and extracellular acidification rate (ECAR). Cells were plated at a density of 20,000 cells/well and incubated in a 37°C/5% CO_2_ incubator for 24 hours in XF96 cell culture plate. The cartridge plate was hydrated with XF calibrant buffer and incubated overnight (37°C, CO2-free). Prior to the XF measurement, growth medium was exchanged with XF assay medium (XF base medium containing 10 mM glucose, 2 mM L-glutamine and 1 mM pyruvate and 5 mM HEPES). Cells were counted after the assays for optimization. The OCR and ECAR were then adjusted to per 1000 cells for comparison between cell groups. Mitochondrial inhibitors oligomycin 2 μM, 1 μM FCCP and 2 μM antimycin/rotenone (Sigma) were used for the assay.

### Flow cytometry

For cell cycle and γ-H2AX flow cytometry experiments, cells were seeded in 6-well plates 24 hours prior to drug treatment. Drugs were then administered at indicated doses, and the cells were harvested for analysis 24- and 48-hours post-treatment. Cells were fixed in ice-cold 70% ethanol overnight at −20°C, and subsequently stored for up to a week in fixative at −20°C. After cells were washed with PBS (1% BSA), they were incubated with Alexa Fluor^®^ 647 γ-H2AX antibody (#613408, BioLegend) for 1 hour at room temperature. Cells were then washed and stained with RNAse/PI buffer (BD Biosciences #550825).

### RNA-seq data processing and analysis

RNA from RENCA Parental, Sdhb-KO and Fh1-KO was isolated using RNeasy microcolumns (Qiagen) and quantified using NanoDrop. Total RNA quality was determined by estimating the A260/A280 and A260/A230 ratios by NanoDrop. RNA integrity number (RIN) was determined by running an Agilent Bioanalyzer gel. Samples with RIN values of 7 or greater are recommended for library prep. RNA-seq was performed using 100 bp paired-end sequencing on an Illumina NovaSeq6000 according to Illumina protocols. RNA-seq reads were aligned using the nf-core [39] pipeline. Fastq reads were aligned to the mm10 genome using the Hisat2 aligner [40] and read counts were generated using the featureCounts v2.0.3 [41]. DESeq2 v1.30.1 R Bioconductor package [42] was used to identify differentially expressed genes (DEGs) using the alternative shrinkage estimator ashr [42] to control for false discovery rates (FDR), and effect sizes. Differentially expressed gene was determined with a cutoff of 1 for actual fold change and an FDR cutoff of (adjusted *p*-value < 0.05). Gene enrichment analysis was performed with clusterProfiler v3.18.1 R Bioconductor package [18] with strict false discovery rate (FDR) adjusted *p*-value cutoff of < 0.05 to interrogate the gene ontology (GO) database, while pathway analysis was conducted using ReactomePA v1.34.0 [43] to interrogate the Reactome database, using an adjusted *p* value. Values of *p* < 0.05 were accepted at significant. Two biological replicates were performed for each cell lines.

### Whole genome bisulfite sequencing

DNA was obtained from cultured cells using Maxwell RSC (Promega). Genomic DNA was sonicated to an average size of 200 bp using Covaris M220 (Covaris). DNA fragments were end-repaired, adenylated, and ligated to Illumina-compatible adaptors using BIOO NEXTflex Bisulfite-Seq Kit (Perkin Elmer). Bisulfite conversion was performed using EZ DNA Methylation-Gold™ Kit (Zymo Research Corporation) according to the manufacturer’s instructions. Then PCR was performed to enrich bisulfite converted and adaptor-ligated fragments. The resulting libraries were pooled and sequenced. The identification of differentially methylated regions was performed using SeqMonk (The Babraham Institute). To identify locus-specific differences in methylation, we performed Chi-square test with FDR adjusted *p*-values less than 0.05 as well as a logistic regression under the same constraints. We then applied a threshold of 25% absolute difference in methylation between parental and KO cells. Two biological replicates were performed for each cell lines.

### Clonogenic assays

Single-cell suspensions of exponentially growing cultures were seeded into 6-well plates in a range of 1 × 10^2^ to 1 × 10^4^ cells and allowed to adhere. At the time of the seeding, the drug was added in culture medium at indicated doses. Cells were then incubated at 37°C for 8 to 14 days, the 6-well plates fixed and stained simultaneously using 20% methanol containing 0.5% crystal violet (Sigma Aldrich). Colonies were counted and then normalized to the number of cells plated.

### Immunohistochemistry

Immunostaining for Fh1, Sdhb and 2SC was performed on FFPE sections. The evaluation was performed by two pathologists in a single-blind manner (HHY and RRH). We used a commercially available primary rabbit anti-Human FH polyclonal antibody (1:800 dilution, Abcam ab95950), rabbit anti-Human SDHB (1:250 dilution, Sigma Aldrich HPA002868) and rabbit anti-Human 2SC polyclonal antibody (1:1000 dilution, Discovery Antibodies, crb2005017e) with a modified Agilent FLEX Envision detection system to run all steps in Automated Autostainer AS48Link. All FFPE slides were pretreated with Heat Induced Epitope Retrieval (HIER) in Dako PTLink using Envision FLEX Target Retrieval solution—Low pH (6.0) for FH, SDHB and high pH (9.0) for 2SC—and incubated 97°C for 20 minutes. Fh1 staining in allograft cells was considered negative in the presence of an internal positive control in immune or stromal cells or non-tumoral kidney parenchyma. 2SC staining was considered positive when there was 2SC-negative staining in the adjacent non-neoplastic cells. Flex Rabbit Negative Control Immunoglobulin fraction staining, instead of primary antibodies to Fh1 and 2SC, was also performed in parallel with experimental slides for each run as a staining negative control, for which all staining came out all clearly negative.

### *In vivo* efficacy studies

All animal studies were approved by the UCLA and Yale Institutional Animal Care and Use committee and carried out in accordance with regulations in the National Research Council Guide for the Care and Use of Laboratory Animals. The RENCA tumor cell line is a well-established, syngeneic murine renal adenocarcinoma model derived from a spontaneously arising tumor in BALB/c mice [44, 45]. Female BALB/c mice were used for all *in vivo* allograft studies. RENCA Fh1 and Sdhb knockout cells were implanted subcutaneously (1 × 10^6^ cells in 0.1 ml of PBS and Matrigel (Corning) 1:1 mixture). Once the tumor size reached approximately 50 mm^3^, mice were randomized into 4 groups (vehicle, monotherapy TMZ, monotherapy BGB-290, combination) so each group had an approximately equal mean tumor volume. Mice were observed daily and treated as indicated. Tumors were measured twice weekly with calipers, and tumor volume was determined with the following formula for ellipsoid volume: π/6 × (length) × (width)^2^.

### Statistical analysis

Data are shown as means ± SEM and were compared with two-sided *t* tests or ANOVA with Bonferroni correction for repeated measures when appropriate. Statistical analyses were carried out in GraphPad Prism. For *in vivo* assays, replicates were defined as individual mice bearing an allograft tumor. *P* values are indicated either directly on figures or using ^*^ *P* < 0.05, ^**^ *P* < 0.01, ^***^ *P* < 0.001, and ^****^ *P* < 0.0001.

## SUPPLEMENTARY MATERIALS











## Abbreviations

FH: Fumarate Hydratase
SDH: Succinate Dehydrogenase
RCC: Renal Cell Carcinoma
KDM4A/B: Lysine-specific demethylase 4A/B
HR: Homologous Recombination
DSBs: DNA double-strand breaks
PARPis: poly ADP-ribose polymerase inhibitors
TMZ: Temozolomide
HLRCC: Hereditary Leiomyomatosis and Renal Cell Cancer
HPGL/PCC: Hereditary Paraganglioma and Pheochromocytoma
BER: Base Excision Repair
LC-MS: liquid chromatography-mass spectrometry
OXPHOS: Oxidative Phosphorylation
OCR: Oxygen Consumption Rate
ECAR: Extracellular Acidification Rate
DEGs: Differentially Expressed Genes
GO: Gene Ontology
BP: Biological Process
2SC: S-(2-succino)-cysteine
FFPE: Formalin-Fixed Paraffin-Embedded
γ-H2AX: Phosphorylated Histone 2A variant H2AX
53BP1: p53-binding protein 1
IF: Immunofluorescence
HED: human equivalent dose
TET: Ten-eleven translocation
PD-1: Programmed Cell Death Protein-1
