# Supplementary material for: Targeting Krebs-cycle-deficient renal cell carcinoma with Poly ADP-ribose polymerase inhibitors and low-dose alkylating chemotherapy
Source: Oncotarget. 2022 Sep 14;13:1054–67. doi: 10.18632/oncotarget.28273 (PMC9477221; doi:10.18632/oncotarget.28273)
Supplement: Supplementary file 1 [file oncotarget-13-28273-s001.pdf]

Targeting Krebs-cycle-deficient renal cell carcinoma with Poly ADP-ribose polymerase inhibitors and low-dose alkylating chemotherapy

SUPPLEMENTARY MATERIALS

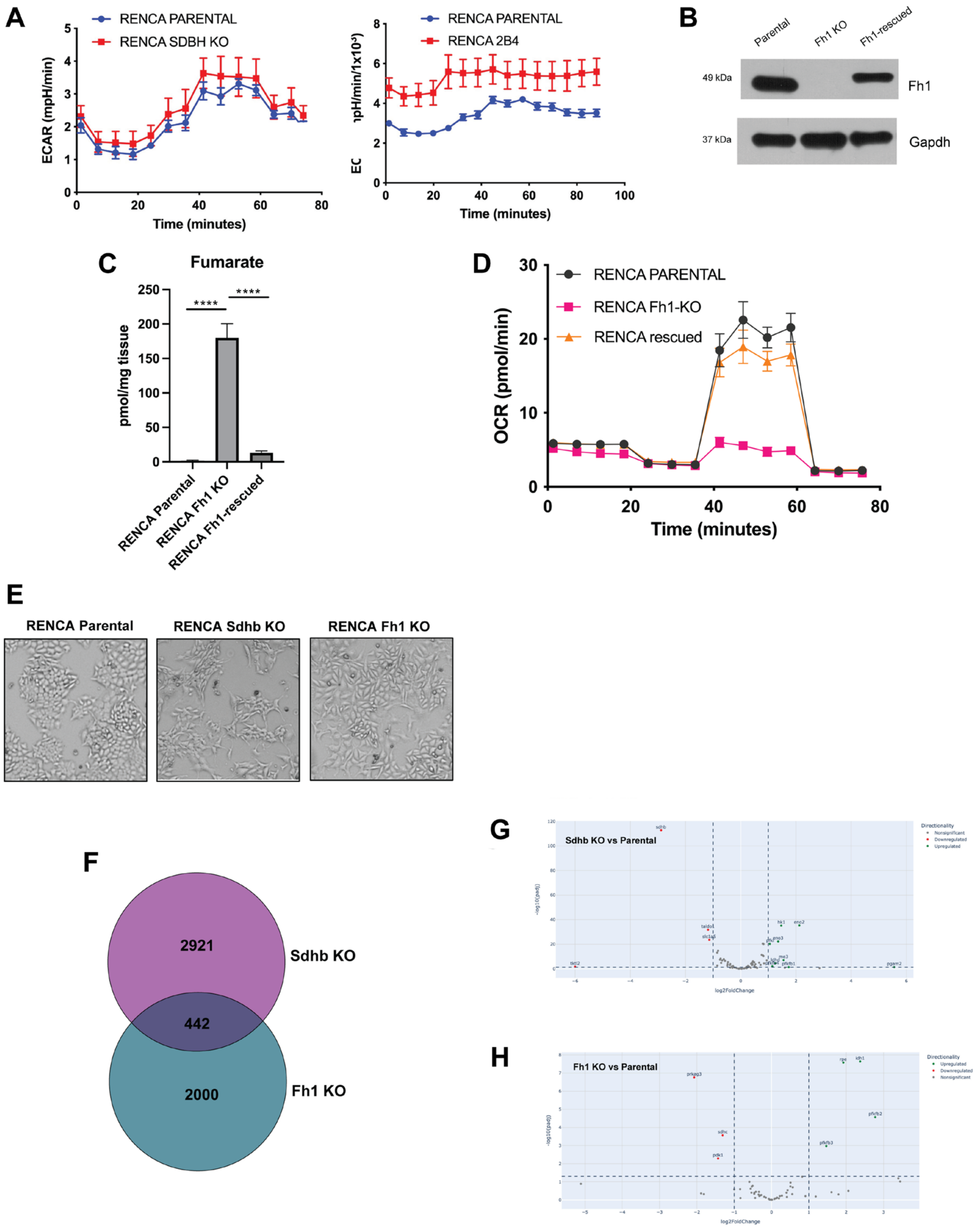

Supplementary Figure 1: (**A**) Seahorse assay showing altered ECAR in Fh1-KO (left panel) and Sdhb-KO (right panel). (**B**) Western blot confirming CRISPR/Cas9 mediated KO of Fh1 and rescued with ORF of Fh1. (**C**) Functional validation of fumarate levels by LC-MS in isogenic cell lines ($n = 3$). (**D**) Seahorse assay showing altered OCR in Fh1-KO and recovered OCR in Fh1-rescued cell line. (**E**) Representative 10× phase contrast images showing altered morphology of Fh1 and Sdhb-deficient cells *in vitro.* (**F**) Venn diagram showing the overlapping differentially expressed genes related to metabolism of Fh1 and Sdhb-deficient cells. (**G**) Volcano plot showing upregulated and downregulated genes of Fh1-deficient cells compared to parental cells. (**H**) Volcano plot showing upregulated and downregulated genes of Sdhb-deficient cells compared to parental cells.

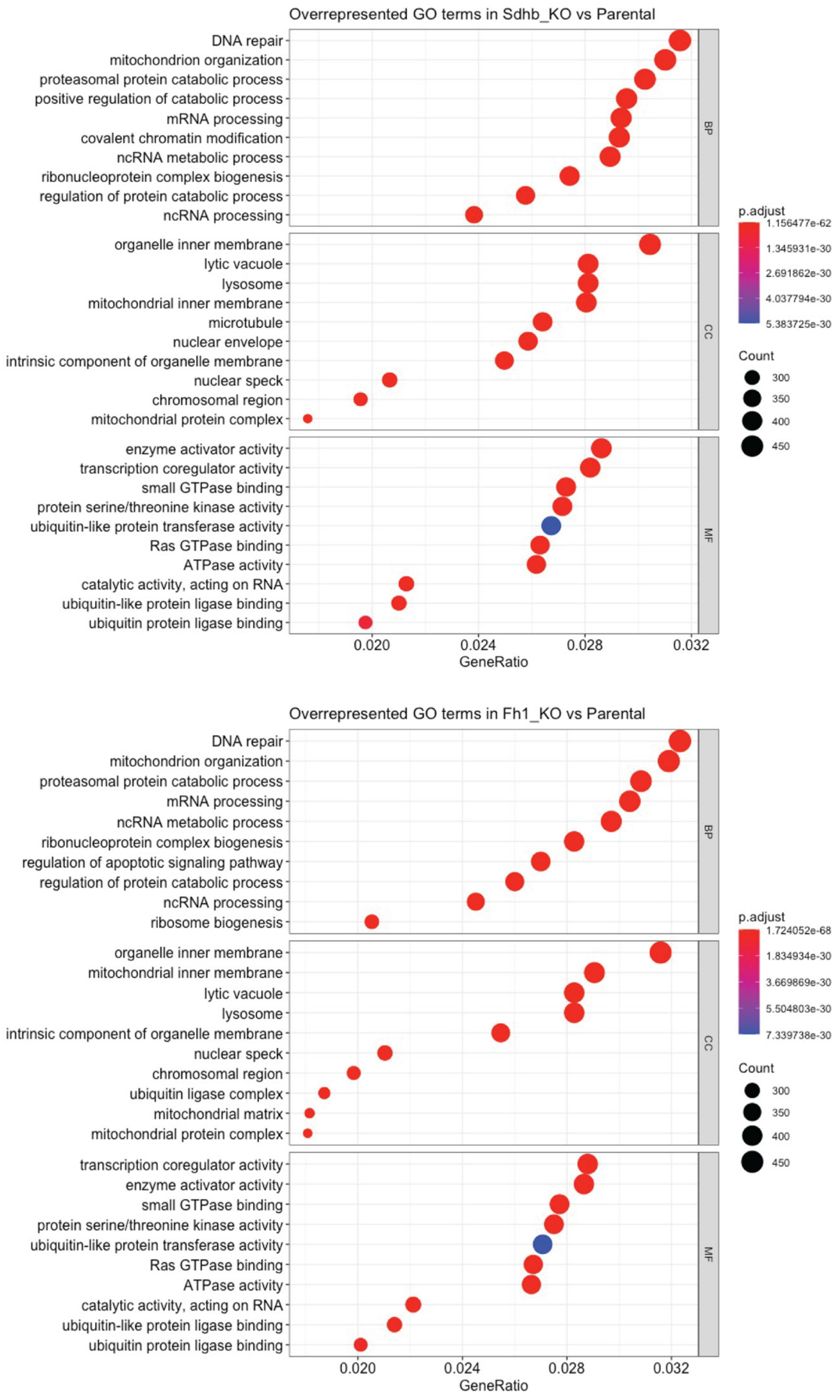

GO = Gene ontology; BP = Biological process; CC = Cellular component; MF = Molecular function

Supplementary Figure 2: Gene ontology (GO) over-representation analysis of Sdhb-KO (top panel) and Fh1-KO DEGs (bottom panel).

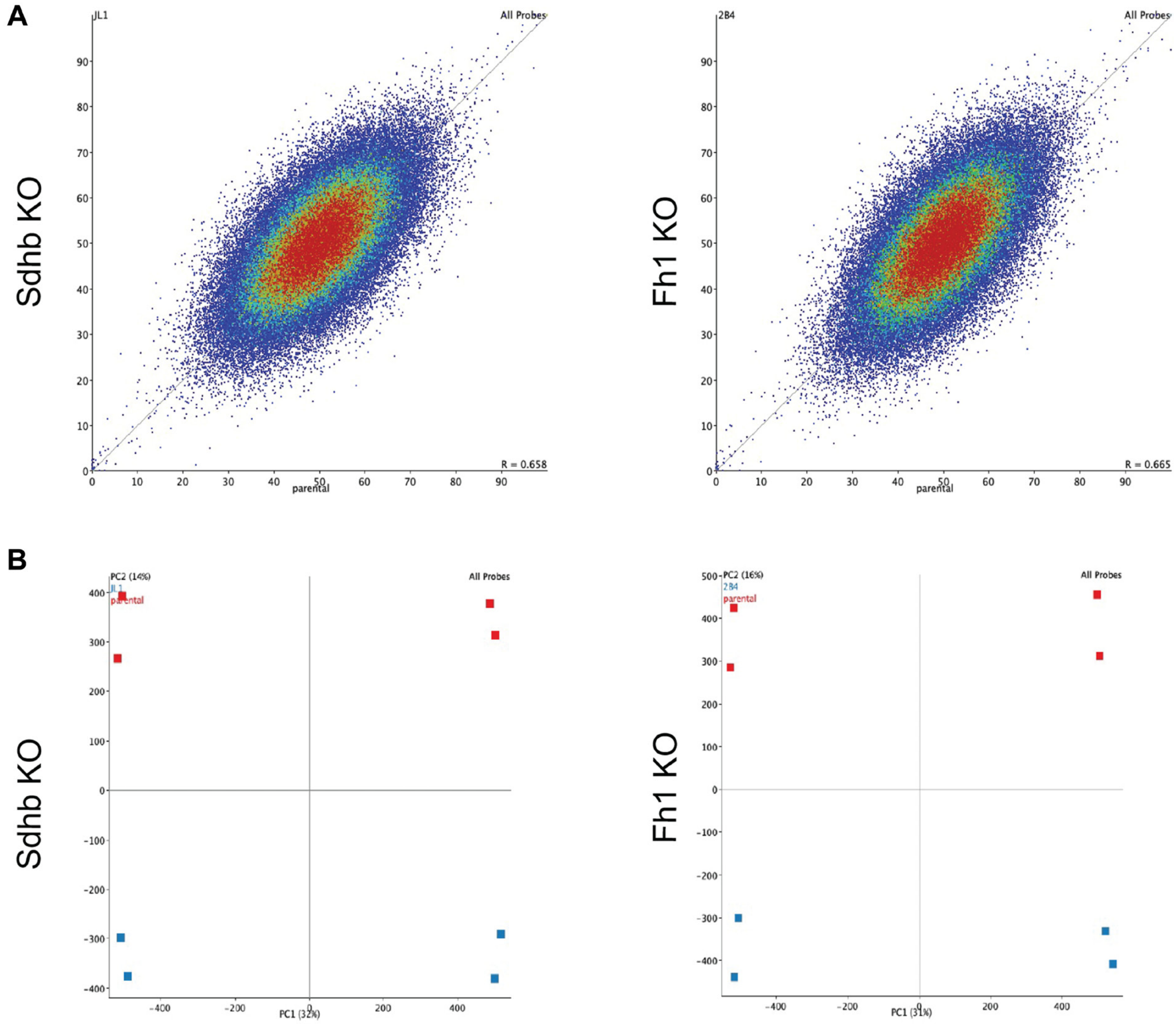

Supplementary Figure 3: (**A**) Global methylation percentage of Fh1-KO and Sdhb-KO compared to the parental cell lines at each quantitated probe. The distribution of the methylation values show that the majority of the genome is between 40% and 60% methylated. (**B**) PCA comparing Fh1-KO and Sdhb-KO to the parental cells.

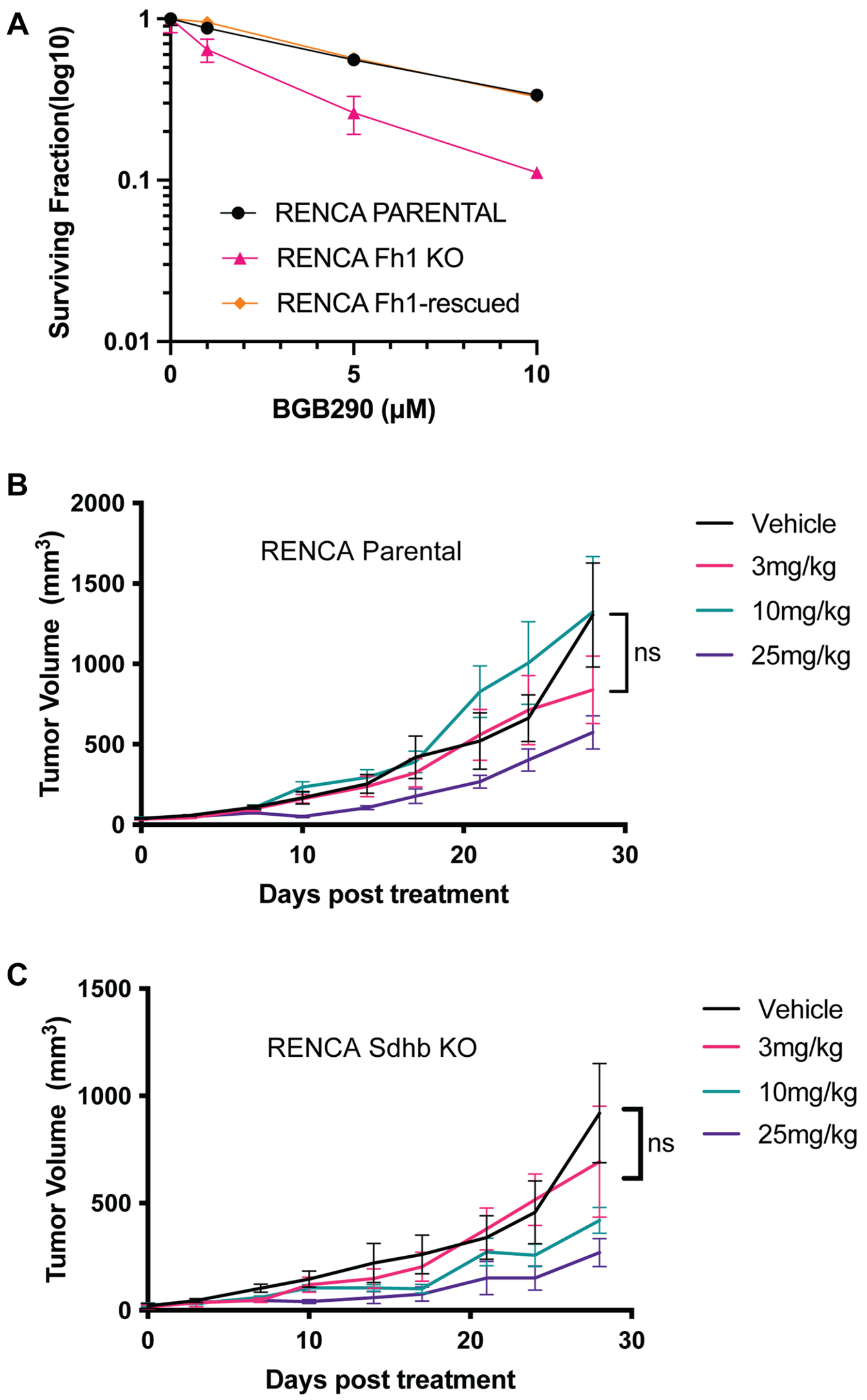

Supplementary Figure 4: (**A**) Quantification of clonogenic survival assays of RENCA parental vs Fh1-KO and Fh1-rescued cells treated with a dose range of BGB-290. (**B**) RENCA parental or (**C**) Sdhb-KO cells were injected subcutaneously into the flank of BALB/c mice. Seven days after injection, tumors were measured and mice were randomized into 4 treatment groups: no treatment (n = 3), TMZ (3 mg/kg) (n = 5), TMZ (10 mg/kg) (n = 5), and TMZ (25 mg/kg) (n = 5). Treatment was initiated when tumors reached an average size of 40–100 mm^3. Mice were treated in 5-day cycles for a total of 3 cycles. Data are represented as mean ± SEM. P values were calculated using two-way ANOVA. $^{***}P < 0.001$, $^{**}P < 0.01$, $^{*}P < 0.05$.

Supplementary Table 1: Differentially expressed genes of Sdhb-KO cells compared to parental. See Supplementary Table 1

Supplementary Table 2: Differentially expressed genes of Fh1-KO cells compared to parental. See Supplementary Table 2

Supplementary Table 3: Differentially methylated genes of Sdhb-KO cells compared to parental. See Supplementary Table 3

Supplementary Table 4: Differentially methylated genes of Fh1-KO cells compared to parental. See Supplementary Table 4

Supplementary Table 5: sgRNA for Sdhb KO

Name	Type	Sequence
SDHB guide RNA top	gRNA Oligo	TTGTGACCTCGAATGCAGACGTACG
SDHB guide RNA bottom	gRNA Oligo	AAACCGTACGTCTGCATTCGAGGT
SDHB gRNA sequencing (Forward)	primer	GTGAACTGTACAAGCTGTGCCA
SDHB gRNA sequencing (Reverse)	primer	TACTGGCATACGTTCCGTAGCA

Supplementary Table 6: sgRNA for Fh1 KO

Name	Type	gRNA target Sequence
sc-420348 A	Plasmid	AATTGGGCGAACTCACACGC
sc-420348 B	Plasmid	CGTGTAGAGTTCGACACCTT
sc-420348 C	Plasmid	ATGCCGAAAGCTTGAATGAC
